# Assessment of Sleep-Related Breathing Disorders in Patients With Duchenne Muscular Dystrophy

**DOI:** 10.4021/jocmr1075w

**Published:** 2012-09-12

**Authors:** Muzaffer Polat, Ozcan Sakinci, Betul Ersoy, Rabia Gonul Sezer, Hikmet Yilmaz

**Affiliations:** a Department of Pediatric Neurology, Celal Bayar University School of Medicine, 45040 Manisa, Turkey; b Resident, Department of Pediatrics, Celal Bayar University School of Medicine, 45040 Manisa, Turkey; c Department of Pediatric Endocrinology, Celal Bayar University School of Medicine, 45040 Manisa, Turkey; d Department of Pediatrics, Zeynep Kamil Maternity and Childrens Diseases Training and Research State Hospital, Uskudar 34668, Istanbul, Turkey; e Department of Neurology, Celal Bayar University School of Medicine, 45040 Manisa, Turkey

**Keywords:** Duchenne muscular dystrophy, Neuromuscular diseases, Obstructive sleep apnea, Polysomnography

## Abstract

**Background:**

Respiratory failure is a significant cause of morbidity and mortality in neuromuscular diseases. Although clinical findings and respiratory function tests aid in diagnosing sleep-related breathing disorders, polysomnography is the gold standard for the diagnosis of these disorders. We aimed to investigate the role of sleep-wake symptoms and clinical findings of patients with Duchenne muscular dystrophy (DMD) in predicting sleep-related breathing disorders through the comparison of polysomnography findings. In addition, we evaluated the sleep architecture of our patients.

**Methods:**

A total of 35 children (12 patients with DMD and 23 controls) were included in this cross-sectional study. Activity status and clinical severity of the patients were determined by history and clinical findings such as scoliosis, obesity. All subjects were hospitalized for one night in the Sleep Unit and their polysomnography examinations were performed. Sleep, breathing, arousals and limb movements were scored manually according to the American Sleep Disorders Association criteria.

**Results:**

Nocturnal and daytime symptoms were present in 50% of patients with DMD, 40.8% were wheelchair-bound and 58% had scoliosis. Obstructive sleep apnea was noted in 16.6% of patients with DMD. The apnea-hypopnea index, leg movement index were significantly higher in the DMD group as compared to the control group (P < 0.05). The number of desaturations, total arousal index and the percentage of total superficial sleep were significantly higher in patients with wheelchair, scoliosis, sleep-wake symptoms.

**Conclusions:**

Being wheelchair-bound or having scoliosis do not predict sleep-related breathing disorders, so patients with DMD should be followed-up via polysomnography. Sleep-wake symptoms should be carefully questioned in these patients and symptomatic patients should be referred to pediatric respiratory units.

## Introduction

Respiratory failure is a significant cause of morbidity and mortality in neuromuscular diseases. Recent advances in respiratory support technologies have increased survival and quality of life in individuals with neuromuscular diseases, Duchenne muscular dystrophy (DMD) in particular [1, 2].

Respiratory failure initially occurs during rapid eye movement (REM) sleep due to atony of the muscles other than the diaphragm. Particularly, weakness of the upper airway dilator muscles may lead to sleep-related breathing disorders such as obstructive sleep apnea (OSA) by increasing airway resistance [3, 4].

Nocturnal ventilatory support is required when a sleep-related breathing disorder is detected. Symptoms due to hypoventilation during sleep should be sought. These symptoms include difficulty in maintaining sleep, snoring, apnea, morning headaches, nonrestorative or unrefreshing sleep, daytime hypersomnolence, lethargy, fatigue, easily getting tired, forgetfulness, attention deficits and concentration problems. However, it has been reported that these symptoms may sometimes be latent or may not necessarily predict sleep-related breathing disorders even if they are detected [5, 6]. Although polysomnography (PSG) is the gold standard for the diagnosis of sleep-related breathing disorders, many other methods to predict sleep-related breathing disorders have been investigated since PSG is not readily available in every center, is expensive and is a difficult-to-perform technique due to social reasons [7].

Our aim was to investigate the role of clinical severity, nocturnal and daytime symptoms in predicting sleep-related breathing disorders in young patients with DMD through the comparison of PSG findings in patients with or without sleep-related breathing disorders. In addition, we evaluated the sleep architecture of our patients.

## Materials and Methods

### Study group

A total of 35 children, 12 patients with DMD followed in the Department of Pediatric Neurology of Celal Bayar University, School of Medicine and 23 controls were included in the study. The diagnosis DMD was based on clinical examination, creatine phosphokinase analysis, electromyography, immunohistochemical examination of muscle biopsy and DNA mutation analysis. The subjects were evaluated for the presence of obesity (assessed by using body mass index (BMI)) and scoliosis (classified as mild < 30^o^, moderate = 31 - 50^o^, severe > 51^o^), both of which might lead to respiratory problems. Nocturnal and daytime symptoms of sleep-related breathing disorders were sought. These symptoms included snoring, sleep apneas observed by parents, abnormal movements during sleep, inadequate and interrupted sleep, morning headache, hypersomnia, lethargy, daytime dyspnea and chest pressure.

### Control group

PSG findings of 23 young children who participated in another study conducted in the Department of Pediatric Endocrinology and Metabolism of Celal Bayar University, School of Medicine, who were aged between 7 and 14 years, who did not have any systemic diseases or respiratory problems were used.

### Polysomnography

All subjects were hospitalized for one night in the Sleep Unit of the Department of Neurology in Celal Bayar University School of Medicine, and their PSG examinations were performed. Sleep data was stored on a computer using PSG digital system (Embla N7000, Medcare, Iceland). These data were then assessed by the same neurologist (HY), specialized in PSG. Sleep architecture was scored visually, according to the criteria of Rechtschaffen and Kales [8]. Nocturnal PSG included central and occipital electroencephalography with electrodes placed according to the international 10 - 20 system (C3-A2, C4-A1, O1-A2, O2-A1), right and left electrooculography, surface electromyography of the jaw and lower extremity muscles, electrocardiography, assessment of oronasal airflow, respiratory movements of the chest and abdomen, oxygen saturation and body position, recording of snoring sounds with a microphone placed over the trachea and simultaneous video recording. Biological calibration was performed in all subjects and artifacts were determined. Sleep, breathing, arousals and limb movements were scored manually according to the American Sleep Disorders Association criteria. The following criteria were used to define apneas and hypopneas: lasting ≥ 10 seconds; > 50% decline in baseline airflow amplitude; and an oxygen desaturation of ≥ 3% [9].

The study was approved by the ethics committee of Celal Bayar University, School of Medicine. Informed parental consent was obtained for each patient.

### Statistical analysis

Statistical analyses were performed using the Statistical Package for the Social Sciences (version 16.0; SPSS Inc., Chicago, IL, USA). A chi-square test was used to compare differences in proportions between two independent groups. A p value of < 0.05 was considered statistically significant. In addition to descriptive statistical methods (mean, standard deviation), the Mann-Whitney U test was used to compare the study groups.

## Results

The mean age of the patients with DMD was 10.33 ± 4.0 years and the mean age of the control group was 10.4 ± 4.2 years. No significant difference was found between the patient and control groups with respect to BMI, age (Table 1).

**Table 1:** Demographic Characteristics of the Patients and the Control Group

	Patient Group mean ± SD	Control Group mean ± SD	P-value
Age (years)	10.33 ± 4.0	10.4 ± 4.2	0.366
Weight (kg)	29.6 ± 1.2	27.4 ± 7.5	1
BMI	16.8 ± 4.6	16.5 ± 1.3	0.366
BMI ± SDS	-1.0 ± 2.6	-0.44 ± 0.99	0.728

SD: standard deviation, BMI: body mass index (weight/height^2^), SDS: standard deviation score.

Seven (58%) patients had scoliosis (2 patients had mild, 3 had moderate and 2 had severe degrees of scoliosis), 5 (40.8%) patients were wheelchair-bound, 50% had nocturnal and/or daytime symptoms. None of the patients had significant adenotonsillar hypertrophy and upper airway abnormalities on examination.

OSA was noted in 2 (16.6%) patients; both of them were wheel-chair bound accompanied with nocturnal and daytime symptoms, 1 patient had moderate degree of scoliosis.

When polysomnographic findings of the patient and control groups were compared, the apnea-hypopnea index and the duration of apnea-hypopnea were found to be significantly higher in the DMD group as compared to the control group (P = 0.012 and P = 0.034, respectively). The leg movement index was significantly higher in the DMD group as compared to the control group (P = 0.019) (Table 2).

**Table 2:** Comparison of the Polysomnographic Findings of Patients With Duchenne Muscular Dystrophy and Controls

	Patient Group (n = 12) mean ± SD	Control Group (n = 23) mean ± SD	P-value
Number of arousals from sleep	34.35 ± 37	45.24 ± 45	0.47
Sleep onset latency	34 ± 30	20.69 ± 13	0.322
Sleep efficiency (%)	89 ± 11	88.98 ± 10	0.80
Percentage of total superficial sleep (N1 N2)	42.51 ± 51	34.83 ± 12	0.18
Percentage of deep sleep (N3)	29.74 ± 7.2	37.16 ± 10	0.317
Percentage of REM sleep	27.63 ± 14	25.59 ± 10	0.414
Leg movement index	16 ± 7.01	11.01 ± 6.2	0.019
Total arousal index	18.85 ± 22	35.95 ± 21	0.07
Apnea-hypopnea index	2.5 ± 2.8	0.75 ± 0.9	0.012
Longest duration of apnea-hypopnea	35.75 ± 28	15.54 ± 16	0.034
Mean duration of apnea-hypopnea	17.01 ± 6.3	10.78 ± 8.7	0.10
Lowest oxygen saturation	92.66 ± 2.9	90.56 ± 4.5	0.214
Number of desaturations	3.83 ± 8.5	0.95 ± 1.8	0.18

REM: rapid eye movement.

The number of desaturations and the total arousal index was significantly higher in DMD patients with scoliosis as compared to those without (P = 0.003 and P = 0.007, respectively). As for sleep stages, the percentage of REM sleep and the sleep efficiency were significantly lower in DMD patients with scoliosis (P = 0.042 and P = 0.019, respectively), while the duration of superficial sleep and the percentage of total superficial sleep were significantly higher in DMD patients with scoliosis (P = 0.042 and P = 0.012, respectively).

While the number of desaturations and the total arousal index was significantly higher (P = 0.008 and P = 0.012, respectively), the duration of REM sleep was significantly lower (P = 0.028) in DMD patients using wheelchair (non-ambulatory) as compared to those who were not using wheelchair (ambulatory). Although the mean duration of apnea-hypopnea was higher in ambulatory patients as compared to non-ambulatory patients, the difference was not significant (P = 0.051).

When DMD patients with sleep-wake symptoms were compared with controls, the apnea-hypopnea index, number of desaturations and the longest duration of apnea-hypopnea were found to be significantly higher in the DMD group with sleep-wake symptoms as compared to the control group (P = 0.024, P = 0.005 and P = 0.026, respectively). The sleep onset latency, percentage of deep sleep, duration of deep sleep and the percentage of total superficial sleep were found to be significantly lower in the DMD group with sleep-wake symptoms as compared to the control group (P = 0.038, P = 0.002, P = 0.012 and P = 0.013, respectively).

The number of desaturations, total arousal index and the percentage of total superficial sleep were noted to be significantly higher in DMD patients with sleep-wake symptoms compared to those without (P = 0.005, P = 0.004 and P = 0.001, respectively).

## Discussion

Sleep-related breathing disorders are significant causes of morbidity and mortality in neuromuscular diseases. Disorders of pulmonary gas exchange and sleep architecture have been reported in as high as 80% of the patients with neuromuscular diseases [10, 11].

While OSA occurs in the first decade in neuromuscular diseases, especially in DMD, nocturnal hypoventilation occurs in the second decade [3]. None of the patients in our study had nocturnal hypoventilation. This was an expected finding as the patients enrolled in this study were in a young age group. Obesity, significant tonsillar hypertrophy or upper airway abnormalities were not found in any of the patients in our study.

In a study involving patients with DMD, OSA was reported in 30%. Most of these patients were wheelchair-bound. The authors did not found a relationship between sleep-wake symptoms and pulmonary function tests, and sleep-related breathing disorders, but recommended PSG for wheelchair-bound patients [3]. Several other studies have also failed to find an association between daytime symptoms and sleep-related breathing disorders [6, 7, 12]. OSA was found in 16.6% of DMD patients included in this study, both of the patients were wheelchair-bound. The low incidence of OSA may be related to the very young age of patients in the study group.

A study reported increased apnea-hypopnea index and decreased oxygen saturation during REM sleep, but these findings were not found to be associated with sleep-related symptoms and daytime gas exchange [4, 13]. Similar to the above-mentioned studies, we also found that apnea and hypopnea were more common during REM sleep.

We did not find a significant difference between ambulatory DMD patients and wheelchair-bound patients with respect to sleep-related breathing disorder indicating parameters such as apnea-hypopnea index and oxygen desaturation and lowest oxygen level. However, the two DMD patients who were found to have an apnea-hypopnea index of > 5 were using wheelchair. In addition, being wheel-chair bound was shown to distrupt sleep architecture, decrease REM sleep and increase arousal index.

The development and progression of scoliosis are expected to reduce the vital capacity by contributing to restrictive pulmonary problems [14, 15]. It has been suggested that spinal corrective surgery does not increase the vital capacity in DMD patients with scoliosis, but improves quality of life and facilitates nursing care rather than preserving pulmonary functions [16]. In this study, as far as sleep quality is concerned, the sleep efficiency, duration of REM sleep were found to be significantly low and the arousal index, superficial sleep duration were found to be significantly high in patients with scoliosis.

In the current study, the presence of scoliosis in DMD patients was not found to have any influence on the apnea-hypopnea index and lowest oxygen saturation. However, the number of desaturations was significantly higher in DMD patients with scoliosis compared to those without. Also, one of the two DMD patients, who were found to have OSA, had scoliosis while the other did not. We could not compare the relationship between the severity of scoliosis and OSA, since only one patient with moderate degree of scoliosis had OSA.

A study reported the prevalence of nocturnal and daytime symptoms caused by sleep disorder as high as 42% in DMD patients with moderate and severe sleep-related breathing disorders, however, these symptoms were not found to have a predictive value [17]. Other studies have also reported that daytime symptoms did not reflect sleep-related respiratory problems in DMD patients [5-7, 12].

In this study, sleep-wake symptoms were found in 50% of DMD patients. However, there were no significant differences between symptomatic and asymptomatic patients with respect to apnea-hypopnea index, thus sleep-related breathing disorders. On the other hand, the apnea-hypopnea index, number of desaturations and the longest duration of apnea-hypopnea were found to be significantly higher in DMD patients with nocturnal and daytime symptoms as compared to the control group, while the apnea-hypopnea index was not significantly higher in asymptomatic DMD patients as compared to the control group. When we evaluated sleep architecture of symptomatic patients, duration of superficial sleep and arousal index were higher as expected.

As in previous studies, all of these findings showed that symptomatology in DMD patients were highly sensitive but not specific. When the patient and control groups were compared, parameters indicating sleep-related breathing disorders such as apnea-hypopnea index, number of desaturations and the longest duration of apnea-hypopnea were found to be higher in the patient group. Therefore, we may suggest that polysomnographic evaluation is indicated for DMD patients, particularly for symptomatic ones.

Nocturnal non-invasive intermittent positive pressure ventilation is an effective treatment and improves quality of life and survival by 5 - 10 years [18]. Patients with neuromuscular disease with nocturnal hypoventilation are likely to deteriorate with the development of daytime hypercapnia and/or progressive symptoms within 2 years and may benefit from the introduction of nocturnal non-invasive ventilation before daytime hypercapnia ensues [19]. Therefore, two DMD patients, who were found to have sleep-related breathing disorders, were started on nocturnal non-invasive intermittent positive pressure ventilation.

The most important aspect of our study was the comparison of PSG findings of the patients with that of the controls. To the best of our knowledge, most of the studies on this issue were conducted either for the detection of sleep-related breathing disorders in neuromuscular diseases via PSG or for improving survival and quality of life via respiratory support in the early period. We were able to compare polysomnographic findings of DMD patients and controls. One limitation of our study may be the number of patients included in the study.

Despite the low number of cases, we concluded that being wheel-chair bound and/or having scoliosis distrups sleep architecture, causes nocturnal and daytime symptoms and decreases quality of life in DMD patients. Larger scaled studies evaluating not only sleep related respiratory diseases, but also the effect of respiratory support on quality of life in patients with disturbance of sleep architecture are needed.

PSG is the gold standard for the diagnosis of sleep-related breathing disorders in neuromuscular diseases. Since clinical indicators such as becoming wheelchair-bound and scoliosis do not predict sleep-related breathing disorders, this group of patients should be followed-up by PSG. Also, sleep-wake symptoms should be carefully questioned in these patients and symptomatic patients should be referred to pediatric respiratory units.
